# Prevalence of Anemia and Associated Factors among Pregnant Women in North Western Zone of Tigray, Northern Ethiopia: A Cross-Sectional Study

**DOI:** 10.1155/2015/165430

**Published:** 2015-06-07

**Authors:** Abel Gebre, Afework Mulugeta

**Affiliations:** ^1^Department of Public Health, College of Health Sciences, Samara University, Samara, Ethiopia; ^2^Department of Public Health, College of Health Sciences, Mekelle University, Mekelle, Ethiopia

## Abstract

*Background*. Anemia affects the lives of more than 2 billion people globally, accounting for over 30% of the world's population. Anemia is a global public health problem occurring at all stages of the life cycle but the burden of the problem is higher in pregnant women particularly in developing countries. The aim of this study was to determine the prevalence of anemia and associated factors among pregnant women attending antenatal clinics in north western zone of Tigray, northern Ethiopia. *Methods*. A facility based cross-sectional study was employed. A systematic random sampling procedure was employed to select 714 pregnant women who were attending antenatal clinics in health facilities found in the study area from April to May 2014. The data was entered and analyzed using Epi-info version 3.5.1 and SPSS version 20.0 statistical software, respectively. Logistic regression analysis was used to identify factors associated with anemia among the study participants. All tests were two-sided and *p* value < 0.05 was considered statistically significant. *Results*. The overall prevalence of anemia (hemoglobin < 11 g/dL) among the pregnant women was 36.1% (95% CI = 32.7%–39.7%) of which 58.5% were mildly, 35.7% moderately, and 5.8% severely anemic. In pregnant women, rural residence (AOR = 1.75, 95% CI = 1.01–3.04), no education/being illiterate (AOR = 1.56, 95% CI = 1.03–2.37), absence of iron supplementation during pregnancy (AOR = 2.76, 95% CI = 1.92–5.37), and meal frequency of less than two times per day (AOR = 2.28, 95% CI = 1.06–4.91) were the independent predictors for increased anemia among the pregnant women. *Conclusions*. Anemia was found to be moderate public health problem in the study area. Residence, educational status, iron supplementation during pregnancy, and meal frequency per day were statistically associated with anemia among the pregnant women. Awareness creation and nutrition education on the importance of taking iron supplementation and nutritional counseling on consumption of extra meal and iron-rich foods during pregnancy are recommended to prevent anemia in the pregnant women.

## 1. Background

Anemia affects the lives of more than 2 billion people globally, accounting for over 30% of the world's population which is the most common public health problem particularly in developing countries occurring at all stages of the life cycle [1, 2]. The prevalence of anemia in developing and developed countries is estimated to be 43% and 9%, respectively [3].

Anemia in pregnant women remains one of the most intractable public health problems in developing countries because of various sociocultural problems like illiteracy, poverty, lack of awareness, cultural and religious taboos, poor dietary habits, and high prevalence of parasitic infestation. Current estimates from the World Health Organization (WHO) put prevalence of anemia at 41.8% among pregnant women, with the highest prevalence rate (61.3%) found among pregnant women in Africa and 52.5% among South East Asia [4–6]. Sub-Saharan Africa is the most affected region, with anemia prevalence among pregnant women estimated to be 17.2 million, which corresponds to approximately 30% of total global cases [7]. Globally, anemia contributes to 20% of all maternal deaths. Anemia in pregnancy may also lead to premature births, low birth weight, fetal impairment, and infant deaths [4, 8, 9].

Anemia during pregnancy is defined by the Centers of Disease Control and Prevention (CDC) and World Health Organization as a hemoglobin concentration less than 11 g/dL. It is considered severe when hemoglobin concentration is less than 7.0 g/dL, moderate when hemoglobin falls between 7.0 and 9.9 g/dL, and mild when hemoglobin is from 10.0 to 10.9 g/dL [10–12]. According to the Ethiopian Demographic and Health Survey (EDHS) report, 17% of reproductive age women are estimated to be anemic and 22% of the pregnant women are anemic [13]. The contextual factors contributing for anemia among pregnant women are different. Interaction of multiple factors like women's sociodemographic, economic, nutritional, and health related factors causes anemia in pregnant women. The availability of local information on the magnitude and related risk factors has a major role in the management and control of anemia in pregnancy. However, there is no adequate and reputable information on the prevalence and factors leading to anemia in pregnant women in Ethiopia and the study area in particular. Therefore, the aim of this study was to determine the prevalence of anemia and associated factors among pregnant women attending antenatal clinics in north western zone of Tigray, northern Ethiopia.

## 2. Methods 

### 2.1. Study Setting and Design

A facility based cross-sectional study was conducted from April to May 2014 in randomly selected health facilities found in north western zone of Tigray which is located at 1087 Kilometer from Addis Ababa and 304 Kilometer from Mekelle, the regional capital city. According to the 2007 national census, the total estimated pregnant women in study area are 25,052. There are two governmental hospitals and twenty-eight public health centers which provides routine antenatal care service to the community [14].

### 2.2. Study Population

All pregnant women attending antenatal care in the governmental health facilities in the study area were target for the study. The study population consisted of a sample of pregnant women who were residing in the study area during the study period and attending heath facilities found in the study area. Those pregnant women who were not long-term residents of the study area (less than 6 months) were excluded. All pregnant women were excluded from the study if they have any of the following disorders including being seriously ill, mental disorder, and women who are unable to hear and/or speak during data collection period.

### 2.3. Sample Size and Sampling Procedures

Sample size was determined based on the single population proportion formula using **Z** ^2^ × **p** × **q**/**d** ^2^ with a 95% CI, 5% margin of error, and an assumption that 31.6% of pregnant women are anemic in the study area [15]. Assuming a 10% nonresponse rate and a design effect of 2, a total sample size of 731 pregnant women was required. Multistage sampling technique was used to select the study participants. One hospital and twelve health centers which provide routine antenatal care services for the pregnant women were selected using a lottery method. A proportional allocation was employed to obtain the sample size from the selected health facilities and a systematic random sampling method was used to select the study participants from each antenatal clinic in the respective health facilities during the data collection period.

### 2.4. Data Collection

Data was collected using pretested interviewer administered questionnaire, which contains sociodemographic characteristics (age, education, occupation, marital status, and others), obstetric and gynecological history (trimester, gravidity, parity, and others), and dietary factors (iron intake, meal frequency, intake of coffee or tea, etc.). Blood hemoglobin concentration was measured using a HemoCue Hb 301 analyzer (manufactured by Ängelholm, Sweden), a precalibrated instrument designed for the measurement of hemoglobin concentration. Venous blood was drawn, through microcuvettes, and inserted into the HemoCue Hb analyzer and the result was recorded.

### 2.5. Statistical Analysis

Data were analyzed using SPSS version 20 after the data were entered to Epi-info version 3.5.1 and exported to it. Categorical variables were summarized as numbers and percentages, whereas normally distributed continuous variables were presented as means and standard deviations. To identify factors associated with the outcome variable (anemia), first a bivariate logistic regression analysis was performed for each independent variable and crude odds ratio (COR) with 95% confidence intervals was obtained. Then, significant variables observed in the bivariate logistic regression analysis (*p* value < 0.2) were subsequently included in the multivariable logistic regression model to determine independent predictors for the outcome variable among the pregnant women. The strength of statistical association was measured by adjusted odds ratios (AOR) and 95% confidence intervals. All tests were two-sided and *p* value < 0.05 was considered statistically significant. The goodness of fit of the final logistic model was tested using Hosmer and Lemeshow test at a *p* value > 0.05.

### 2.6. Ethical Considerations

The study was conducted after getting ethical clearance from Mekelle University, College of Health Sciences, Institutional Review Board (IRB). Support letter was obtained from Tigray Regional Health Bureau and concerned health departments. Written informed consent was secured from study participants after explaining about the objective and purpose of the study to each study participants. The participants were also assured about the confidentiality of the data. While assessing anemia status, the result of the test was communicated immediately to each participant and if the pregnant woman was anemic, she was referred to the health personnel for treatment and follow-up.

## 3. Results

### 3.1. Socioeconomic and Demographic Characteristics of the Pregnant Women

A total of 714 study participants were included in the study making a response rate of 97.7% (Table 1). The mean age (±SD) of the study participants at present and at first marriage was 25.8 ± 5.84 and 18.2 ± 3.41 years, respectively. Majority (97.3%) of the study participants were currently married. Two-thirds of the study participants' occupations were housewife. The majority (91.7%) of the participants were Tigray in ethnicity followed by Amhara (6.4%). 47.4% of the study participants were in the age range of 25 to 34 years. The majority (90.0%) of the study participants were Orthodox Christian followers.

### 3.2. Obstetric and Nutrition Related Characteristics of the Pregnant Women

The mean current gestational age (±SD) of the study participants was 26.7 ± 8.05 weeks (Table 2). Above two-thirds of the participants were multigravida. More than half (52.2%) of the study participants were in their third trimester. Two-thirds of the participants did not have iron supplementation during pregnancy.

### 3.3. Prevalence of Anemia among the Pregnant Women

**Figure 1:**
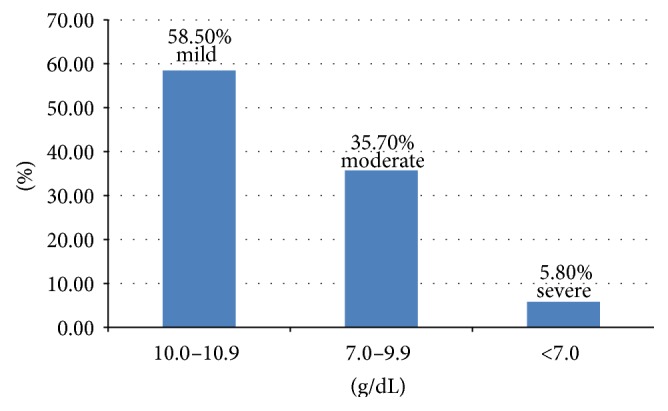
Percentage of anemia by severity among anemic pregnant women (*n* = 258).

The overall prevalence of anemia in this study was 36.1% (95% CI = 32.7%–39.7%). The mean ± SD hemoglobin concentration among the study participants was 11.21 ± 1.18 g/dL. Of the anemic pregnant women, 151 (58.5%), 92 (35.7%), and 15 (5.8%) had mild anemia (Hb ranges 10.0–10.9 g/dL), moderate anemia (Hb ranges 7.0–9.9 g/dL), and severe anemia (Hb < 7.0 g/dL), respectively (Figure 1).

### 3.4. Factors Associated with Anemia among the Pregnant Women

The comparison between the profiles of the pregnant women who had anemia and who did not from the bivariate logistic regression analysis revealed that marital status, residence, educational status, family monthly income, number of visits, age of the women at first marriage, body mass index, iron supplementation, meal frequency per day, and nutrition education were significantly associated with maternal anemia (Table 3). However, in the multivariable logistic regression analysis level after controlling the effect of confounders revealed that variables that were independent predictors for maternal anemia among the pregnant women were maternal residence (AOR = 1.75, 95% CI = 1.01–3.04), educational status (AOR = 1.56, 95% CI = 1.03–2.37), iron supplementation during pregnancy (AOR = 3.76, 95% CI = 1.92–8.37), and meal frequency per day (AOR = 2.18, 95% CI = 1.06–4.91) (Table 3).

## 4. Discussion

Pregnant women are one of the vulnerable groups of a population to develop anemia particularly in developing countries [16]. Therefore, the aim of this study was to determine the prevalence of anemia and associated factors among pregnant women attending antenatal clinics found in north western zone of Tigray, Ethiopia.

This study found that 36.1% (95% CI = 32.7%–39.7%) of the pregnant women in the study area were anemic. According to the World Health Organization classification of the public health importance of anemia, the magnitude indicates that there is moderate public health significance of anemia among the pregnant women in the study area [11, 12].

The prevalence of anemia obtained in this study is almost consistent with other studies conducted among pregnant women attending antenatal clinics in Sidama [15], West Arsi [17], and northern Nigeria [18], with the prevalence of 31.6%, 36.6%, and 30%, respectively. The result of this study was much lower than the previous studies conducted among pregnant women attending antenatal clinics in Gode town [19], north Bengal [20], Udupi district [21], Pakistan [22], Bangladesh [23], and West Bengal [24] with the prevalence of 56.8%, 82%, 50.1%, 90.5%, 73%, and 67.8%, respectively, but higher than a study conducted in Mekelle [3] and Addis Ababa [25] and the national prevalence of anemia noted in 2011, Ethiopian Demographic and Health Survey report [13], where the prevalence anemia among the pregnant women was found to be 11%, 21.3%, and 22%, respectively. Socioeconomic and geographical variations might be the reasons for the different prevalence of anemia among pregnant women across countries and regions. Using different cutoff points and hemoglobin measurement for anemia and study areas may also result in varied prevalence of anemia in pregnant women.

In this study, the multivariable logistic regression analysis revealed that maternal residence, educational status, iron supplementation during pregnancy, and meal frequency per day were significantly associated with anemia among the pregnant women at *p* value ≤ 0.05 (Table 3). However, maternal marital status, family monthly income, number of visits, age of the women at first marriage, body mass index, and nutrition education showed significant association with bivariate analysis but not with the multivariable analysis (Table 3).

In the present study, the prevalence of anemia was higher among pregnant women who were from rural areas as compared to pregnant women residents of urban areas in which the risk of developing anemia among rural pregnant women was 1.75 times higher to be anemic as compared to those pregnant women living in urban areas (AOR = 1.75, 95% CI = 1.01–3.04). This could be due to the reason that pregnant women from rural areas might have lack of information about adequate nutrition during pregnancy, economic factors, and inaccessibility to health care facilities. Similar results were reported by other studies conducted in south eastern Ethiopia (AOR = 3.3, 95% CI = 1.5–7.4) [9], Gondar (AOR = 2.14, 95% CI = 1.51–3.38) [26], and southwest Ethiopia (AOR = 1.62, 95% C.I = 1.02–2.62) [27].

In the present study, the prevalence of anemia was higher among pregnant women who are not educated as compared to those pregnant women who are educated in which pregnant women who were not educated were at 1.56 times higher risk to be anemic as compared to pregnant women who had formal education (AOR = 1.56, 95% CI = 1.03–2.37). The reason for this might be the fact that pregnant women who have some level of formal education can be aware of anemia during pregnancy and take some preventive measures like eating iron-rich food and taking iron tables. The result of this study is consistent with other studies conducted in Addis Ababa (AOR = 2.12, 95% CI = 2.47–6.80) [25], West Bengal (AOR = 17.50, 95% CI = 3.77–90.68) [24], and West Algeria (AOR = 0.79, 95% CI = 0.07–0.49) [28].

In the present study, the prevalence of anemia was higher among pregnant women having a meal frequency of less than 3 times per day as compared to pregnant women who had a meal frequency of more than 2 times per day in which the pregnant women having a meal frequency of less than 3 times per day were at 2 times higher risk of developing anemia as compared to pregnant women who had a meal frequency of more than 3 times per day (AOR = 2.18, 95% CI = 1.06–4.91). This might be due to the reason that pregnancy is a critical period with increased energy and nutrient demand for the mother which should be fulfilled with increased meal frequency per day. This result is consistent with other studies conducted in Mekelle (AOR = 3.88, 95% CI = 1.93, 7.79) [3] and West Arsi (AOR = 4.66, 95% CI = 2.94, 7.38) [17].

In the present study, the prevalence of anemia was significantly higher among pregnant women who did not take iron supplementation during pregnancy as compared to those pregnant women who took their iron supplementation in which pregnant women who did not take iron supplementation were at 3.76 times higher risk to be anemic as compared to pregnant women who took their iron supplementation (AOR = 3.76, 95% CI = 1.92–8.37). The reason for this might be pregnant women who take their iron tablets which can help them to increase their hemoglobin level and prevent anemia during pregnancy time. This result was consistent with other studies conducted in Sidama (AOR = 1.90, 95% CI = 1.14–3.19) [15], Gode town (AOR = 1.54, 95% CI = 1.04–2.27) [19], West Bengal, (AOR = 5.65, 95% CI = 1.78–18.54) [24], and West Algeria (AOR = 0.71, 95% CI = 0.26–0.99) [28].

## 5. Limitation of the Study

There may be a social desirability bias for dietary information and monthly income. This may overestimate the association between the variables and anemia among the pregnant women.

## 6. Conclusion and Recommendations

Anemia was found to be moderate public health problem in the study area. Residence, educational status, iron supplementation during pregnancy, and meal frequency of the woman per day were statistically significant independent predictors for maternal anemia among the pregnant women in the study area.

Awareness creation and nutrition education on the importance of taking iron supplementation and nutritional counseling on consumption of extra meal and iron-rich foods during pregnancy are recommended to prevent anemia in the pregnant women.

## Figures and Tables

**Table 1:** Socioeconomic and demographic characteristics of the pregnant women attending antenatal clinics in north western zone of Tigray, northern Ethiopia, 2014 (*n* = 714).

Variables	*n* (%)	Mean ± SD
Age (years)		
<18	23 (3.2)	25.8 ± 5.84
18–24	288 (40.3)	
25–29	223 (31.2)	
30–34	116 (16.2)	
≥35	64 (9.0)	
Residence		
Urban	401 (56.2)	
Rural	315 (43.8)	
Ethnicity		
Tigray	655 (91.7)	
Amhara	45 (6.4)	
Others	14 (1.9)	
Religion		
Orthodox	643 (90.0)	
Muslim	64 (9.0)	
Others	7 (1.0)	
Marital status		
Married	695 (97.3)	
Divorced	14 (2.0)	
Widowed	3 (0.4)	
Single	2 (0.3)	
Educational status		
Cannot read and write	266 (37.3)	
Can read and write	94 (13.2)	
Primary (grades 1–8)	168 (23.5)	
Secondary (grades 9–12)	137 (19.2)	
Above secondary (above grade 12)	49 (6.9)	
Occupational status		
Housewife	453 (63.4)	
Government employee	219 (30.7)	
Private	30 (4.2)	
Others	12 (1.7)	
Family size		
≤4	511 (71.6)	
5–7	182 (25.5)	
≥8	21 (2.9)	
Age at first marriage (years)		
<18	191 (26.8)	18.2 ± 3.41
≥18	523 (73.2)	
Family income (ETB)		
<500	61 (8.5)	712.4 ± 289.90
500–1000	260 (36.4)	
≥1000	393 (55.0)	

ETB: Ethiopian birr; 1 Ethiopian birr equals 20 USD.

**Table 2:** Obstetric and nutritional characteristics of the pregnant women attending antenatal clinics in north western zone of Tigray, northern Ethiopia, 2014 (*n* = 714).

Variables	*n* (%)	Mean ± SD
Gravidity		
Primigravida	197 (27.6)	
Multigravida	517 (72.4)	
Parity		
Nulliparous	203 (28.4)	
Primiparous	180 (25.2)	
Multiparous	331 (46.4)	
Birth interval (*n* = 511)		
<2 years	232 (45.4)	
≥2 years	279 (54.6)	
Trimester		
First	84 (11.8)	26.7 ± 8.05 weeks
Second	207 (29.0)	
Third	423 (59.2)	
Number of visits		
1 time	169 (23.7)	
2-3 times	434 (60.8)	
≥4 times	111 (15.5)	
Meal frequency per day		
≤2 times	156 (21.8)	
3 times	409 (57.3)	
>3 times	14967 (20.1)	
Iron supplementation		
Yes	236 (33.1)	
No	478 (66.9)	
Malaria infection in the previous one year		
Yes	104 (14.6)	
No	610 (85.4)	
Nutrition education		
Yes	450 (63.0)	
No	264 (37.0)	
Taking tea/coffee immediately after meal		
Yes	420 (58.8)	
No	294 (41.2)	
Body mass index (BMI)		
Low (BMI ≤ 20 kg/m^2^)	156 (21.8)	
Normal (BMI: 20–24.9 kg/m^2^)	149 (20.9)	
High (BMI ≥ 25 kg/m^2^)	409 (57.3)	

**Table 3:** Factors associated with anemia among pregnant women attending antenatal clinics in north western zone of Tigray, northern Ethiopia, 2014 (*n* = 714).

Variables	Anemia		COR (95% CI)	AOR (95% CI)
	Yes (258)	No (456)		
Marital status				
In marital union	247 (35.5%)	448 (64.2%)	1	1
Not in marital union	11 (57.9%)	8 (42.1%)	2.49 (0.99–6.28)	1.23 (0.74–2.00)
Residence				
Urban	126 (31.6%)	275 (68.4%)	1	1
Rural	132 (41.9%)	181 (58.1%)	1.59 (1.15–2.13)	**1.75** (1.01–3.04)^**∗**^
Educational status				
Not educated	160 (44.4%)	200 (55.6%)	2.09 (1.53–2.86)	**1.56** (1.03–2.37)^**∗**^
Educated	98 (27.7%)	256 (72.3%)	1	**1**
Family income (ETB)				
<500	26 (42.6%)	35 (57.4%)	1.82 (0.05–3.16)	0.82 (0.46–1.48)
500–1000	118 (45.4%)	142 (54.6%)	2.03 (2.51–5.57)	1.30 (0.72–2.39)
≥1000	114 (29.0%)	279 (71.0%)	1	1
Age at first marriage (years)				
<18	61 (31.9%)	130 (68.1%)	0.78 (0.55–1.10)	1.03 (0.67–1.58)
≥18	197 (37.7%)	326 (76.8%)	1	1
Number of visits				
1 time	49 (29.0%)	120 (71.0%)	1.55 (0.93–2.57)	1.13 (0.60–2.11)
2-3 times	166 (38.2%)	268 (61.8%)	1.02 (0.67–1.57)	0.93 (0.54–1.59)
≥4 times	43 (38.7%)	68 (61.3%)	1	1
Iron supplementation				
Yes	63 (26.7%)	173 (73.3%)	1	**1**
No	195 (40.8%)	283 (59.2%)	1.89 (1.35–2.66)	**3.76** (1.92–8.37)^**∗**^
Nutrition education				
Yes	146 (32.4%)	304 (67.6%)	1	1
No	112 (42.4%)	157 (57.6%)	1.56 (1.14–2.13)	0.50 (0.22–1.15)
Meal frequency per day				
≤2 times	60 (42.6%)	81 (57.4%)	1.36 (0.79–1.08)	**2.18** (1.06–4.91)^**∗**^
3 times	52 (32.9%)	106 (67.1%)	0.90 (0.75–1.63)	2.31 (0.86–3.39)
>3 times	146 (35.2%)	269 (64.8%)	1	1
Body mass index				
Low (BMI < 20 kg/m^2^)	56 (35.9%)	100 (64.1%)	1.43 (0.88–2.32)	1.59 (0.84–2.98)
Normal (BMI; 20–24.9 kg/m^2^)	42 (28.2%)	107 (71.8%)	1	1
High (BMI ≥ 25 kg/m^2^)	160 (39.1%)	249 (60.9%)	0.87 (0.59–1.28)	0.74 (0.46–1.20)

^*∗*^Statistically significant (*p* < 0.05) 1: reference group; ETB: Ethiopian birr; 1 Ethiopian birr equals 20 USD.

## Abbreviations

AOR: Adjusted odds ratio
CDC: Center of Disease Control and Prevention
COR: Crude odds ratio
CI: Confidence interval
EDHS: Ethiopian Demographic and Health Survey
g/dL: Gram/deciliter
Hb: Hemoglobin
SD: Standard deviation
WHO: World Health Organization.
